# Trajectory of Depression among Victims of Elder Mistreatment

**DOI:** 10.1093/geroni/igaa057.3218

**Published:** 2020-12-16

**Authors:** Gabriella Dong, Mengting Li

**Affiliations:** Rutgers University, New Brunswick, New Jersey, United States

## Abstract

Individuals exposed to elder mistreatment are affected in dissimilar ways. Most existing studies are cross-sectional and fail to capture the change in mental health of older adults with exposure to elder mistreatment. This study aims to examine depression trajectories of elder mistreatment victims and identify protective factors. Data were drawn from the two-wave Population Study of Chinese Elderly in Chicago (PINE) with 725 participants who reported elder mistreatment at the baseline. Depression was measured by Patient Health Questionnaire-9. Self-mastery was assessed by the Pearlin Mastery Scale. Conscientiousness was evaluated by the NEO Five-Factor Inventory. Multinomial logistic regression was used while controlling demographic factors and recurrence of elder mistreatment. We identified four depression trajectories among elder mistreatment victims: chronic (9.61%), delayed (6.27%), recovery resilience (21.17%), and resistance resilience (62.95%). The chronic group was showing severe depression in both waves. The delayed group experienced a delayed reaction with increasing depression over time. The depression level of the recovery resilience group bounced back from elder mistreatment. The resistance resilience group exhibited low depression over time. Elder mistreatment victims with increasing self-mastery were more likely to be in recovery resilience group than in chronic group (RRR=1.05, 95%CI=1.02-1.09). In addition, elder mistreatment victims with increasing conscientiousness were less likely to be in delayed group than in resistance resilience group (RRR=0.96, 95%CI=0.92-1.00). Healthcare providers and social service agents could focus on elder mistreatment victims with chronic and delayed depression trajectories. Interventions could promote mental health of elder mistreatment victims through improving self-mastery and conscientiousness.

